# Synthesis and Antimicrobial Activity of 3-(1,3,4-Oxadiazol-2-yl)quinazolin-4(3*H*)-ones

**DOI:** 10.3797/scipharm.0912-16

**Published:** 2010-04-26

**Authors:** Navin B. Patel, Jaymin C. Patel

**Affiliations:** Department of Chemistry, Veer Narmad South Gujarat University, Surat 395007, Gujarat, India

**Keywords:** Antimicrobial activity, 1,3,4-Oxadiazole, Quinazolin-4(3*H*)-one

## Abstract

In attempt to find new pharmacologically active molecules, we report here the synthesis and *in vitro* antimicrobial activity of various 3-(1,3,4-oxadiazol-2-yl)-quinazolin-4(3*H*)-ones. The antimicrobial activity of title compounds were examined against two gram positive bacteria (*S. aureus*, *S. pyogenes*), two gram negative bacteria (*E. coli*, *P. aeruginosa*) and three fungi (*C. albicans*, *A. niger*, *A. clavatus*) using the broth microdilution method. Some derivatives bearing a bromo or iodo group exhibited very good antimicrobial activity.

## Introduction

The chemistry of heterocyclic compounds has been an interesting field of study for a long time. The synthesis of novel oxadiazole derivatives and investigation of their chemical and biological behavior have gained more importance in recent decades for biological, medicinal and agricultural reasons. 1,3,4-Oxadiazoles represent an important class of heterocyclic compounds. Their derivatives possess a broad spectrum of biological activity in both agrochemicals and pharmaceuticals such as insecticidal, herbicidal, antibacterial, antifungal, analgesic, anti-inflammatory, antimalarial, antiviral, anti-HBV, antianexiety, anticancer, anti-HIV, antitubercular and anticonvulsant [1–14]. Quinazolinone nucleus has been gaining prominence due to the fact that its derivatives have been found to possess wide spectrum of pharmacological properties. Quinazolin-4(3*H*)-one derivatives are useful heterocycles, possessing potent pharmacological activities such as antibacterial, antifungal, analgesic, anti-inflammatory, anthelminthic, anticancer, anticonvulsant, antihistaminic, anti-HIV, antiproliferative, antitubercular, antiviral, CNS depressant, cytotoxicity, diuretic and hypolipidemic [15–30].

1,3,4-Qxadiazoles and quinazolin-4(3*H*)-ones having various heterocycles possess wide range of pharmacological properties. The aim of the present work was to attach 1,3,4-oxadiazole residues to quinazolin-4(3*H*)-one in order to find new biologically active molecules. Thus, the synthesis of novel 1,3,4-oxadiazolyl-quinazolin-4(3*H*)-one derivatives has been achieved.

## Results and Discussion

### Chemistry

The title compounds **9a–f**, **10a–f** and **12a–f** were synthesized according to Scheme 2. The structure of all the synthesized compounds were evaluated by spectral data. Benzoic acid derivatives **1a,b** were converted in to esters **2a,b** using methanol and catalytic amount of sulphuric acid. Esters **2a,b** on treatment with hydrazine hydrate yielded corresponding hydrazides **3a,b**. The IR spectra of **3a,b** showed the absence of ester stretching frequency, instead in gave a band at around 1650 cm^−1^ for carbonyl group and showing sharp bands in the region of 3300–3435 cm^−1^ due to -NHNH_2_ group. Hydrazides on cyclization reaction with methanolic cyanogen bromide and 4-aminobenzoic acid in phosphorus oxychloride yielded amino substituted 1,3,4-oxadiazoles **4a,b** and **5a,b** respectively (Scheme 1). 1,3,4-oxadiazole showed C=N stretching at around 1655 cm^−1^ and C-O-C stretching at around 1270 cm^−1^ and 1040 cm^−1^. Signals at around 158 δ ppm and 164 δ ppm confirmed the C-2 and C-5 carbon of 1,3,4-oxadiazole unite.

Substituted benzoxazinones **8a–c** were prepared by reaction of acid chloride **6** with substituted anthranilic acid **7a–c** in pyridine. Then condensation reaction of **8a–c** with 1,3,4-oxadiazoles **4a,b** and **5a,b** yielded the desired compounds **9a–f** and **10a–f**. Substituted benzoxazinones **8a–c** on reaction with glycine yielded quinazolin-4(3*H*)-ones **11a–c** which on cyclization reaction with hydrazides **3a,b** gave the desired compounds **12a–f** (Scheme 2). IR spectra showed C=O and C=N stretching frequencies of quinazolinone at around 1680 cm^−1^ and 1610 cm^−1^ respectively, further confirmed by ^13^C NMR spectra, which showed C=O and C=N signal at around 160.5 δ ppm and 163.5 δ ppm respectively.

### Antibacterial activity

The minimal bactericidal concentrations of the tested compounds are shown in Tab. 1. The three different series of 1,3,4-oxadiazolyl-quinazolin-4(3*H*)-ones **9a–f**, **10a–f** and **12a–f** were tested for *in vitro* antibacterial activity against two gram positive bacteria (*S. aureus* MTCC 96, *S. pyogenes* MTCC 442) and two gram negative bacteria (*E. coli* MTCC 443, *P. aeruginosa* MTCC 1688). Ampicillin was used as a standard drug. Results showed that, most of the compounds possessed very good antibacterial activity (MBC = 50–250 μg/ml) against gram positive bacteria *S. aureus*. Some of the compounds possessed excellent activity as compared to ampicillin. Compounds **5a**, **9b**, **10a**, **10c**, **10f** and **12c** showed MBC value in the range between 50–150 μg/ml while standard drug ampicillin itself had MBC value of 250 μg/ml against gram positive bacteria *S. aureus*. Compounds **4a**, **4b**, **5b**, **9a**, **9d**, **9e**, **10e** and **12f** imparted parallel activity as ampicillin with MBC in the range 200–250 μg/ml. **5a**, **10c** and **10f** have MBC at 150 μg/ml which were comparatively good as ampicillin against *S. pyogenes*. **4a** and **12f** showed excellent activity at 50 μg/ml while **9c**, **10f**, **12b** and **12e** possessed good activity at 100 μg/ml against gram negative bacteria *E. coli* as compared to ampicillin. Compound **12b** showed excellent activity at 50 μg/ml, while **9e** and **12f** exhibited good activity at 150 μg/ml against *P. aeruginosa*. The remaining compounds of the three different series possessed moderate to poor activities.

### Antifungal activity

Minimal fungicidal concentrations of the synthesized compounds are shown in Table 2. For *in vitro* antifungal activity, three fungal species *C. albicans* MTCC 227, *A. niger* MTCC 282 and *A. clavatus* MTCC 1323 were used and compared with standard drug griseofulvin. Most of the compounds possessed very good antifungal activity against *C. albicans*. Their MFC values are in the range between 100–500 μg/ml. Compound **12c** showed excellent activity at 100 μg/ml whereas compounds **4a**, **9f**, **10b**, **10f**, **12b** and **12f** possessed very good activity at 200–250 μg/ml while **5a,b**, **9a–e**, **10d,e** and **12e** share similar activities as griseofulvin which was 500 μg/ml against *C. albicans*. Compounds **9a**, **9d**, **9f** and **12c** showed moderate activities with MFC of 200–250 μg/ml against *A. niger* and *A. clavatus*. The remaining compounds displayed poor activities against both fungal species *A. niger* and *A. clavatus* as compared to control drug griseofulvin.

## Experimental

### Chemistry

All chemical were of analytical grade and used directly. Melting points were determined in PMP-DM scientific melting point apparatus and are uncorrected. IR spectra were recorded on a Perkin-Elmer RX 1 FTIR spectrophotometer, using potassium bromide pellets and the frequencies are expressed in cm^−1^. The ^1^H NMR and ^13^C NMR spectra were recorded with a Bruker Avance II 400 NMR spectrometer, using tetramethylsilane as the internal reference, with dimethylsulphoxide DMSO-d_6_ as solvent. The chemical shifts are reported in parts per million (ppm). Elemental analyses were performed on a Heraeus Carlo Erba 1180 CHN analyzer. The purity of compounds was confirmed by TLC using Merck silica gel 60 F254 plates using toluene:ethylacetate:methanol (7:2:1) as a mobile phase and spots were visualized under UV radiation. {2-[(2,6-Dichlorophenyl)amino]phenyl}acetyl chloride (**6**) was synthesized by the literature procedure [31].

### General procedure for esters (2a,b)

Substituted benzoic acid **1a,b** (20 mmol) and 30 ml methanol was refluxed on water bath for 5–8 h in a few drops of concentrated sulfuric acid as a catalyst. After completion of the reaction, it was poured onto ice cold water. The obtained solid was washed with sodium bicarbonate solution (5%), dried and recrystallized twice from methanol.

#### Methyl 2-chlorobenzoate (**2a**)

Yield: 75%, bp: 232–236°C. IR (KBr), v, cm^−1^: 740 (C-Cl), 2856, 2960 (CH_3_), 1286, 1121 (C-O-C), 1722 (C=O).

#### Methyl 4-chlorobenzoate (**2b**)

Yield: 72%, mp: 40–45°C. IR (KBr), v, cm^−1^: 742 (C-Cl), 2852, 2959 (CH_3_), 1282, 1119 (C-O-C), 1720 (C=O).

### General procedure for hydrazides (3a,b)

To a solution of methyl benzoates **2a,b** (10 mmol) in 15 ml methanol was added hydrazine hydrate (20 mmol). The reaction mixture was refluxed on a water bath for 8–10 h and allowed to stand overnight. The crystals formed were filtered, washed and after drying recrystallized from methanol.

#### 2-Chlorobenzohydrazide (**3a**)

Yield: 70%, mp: 112–116°C. IR (KBr), v, cm^−1^: 742 (C-Cl), 1650 (C=O), 3308 (NH), 3429, 3348 (NH_2_).

#### 4-Chlorobenzohydrazide (**3b**)

Yield: 67%, mp: 163–166°C. IR (KBr), v, cm^−1^: 745 (C-Cl), 1645 (C=O), 3310 (NH), 3433, 3352 (NH_2_).

### General procedure for 5-substituted phenyl-1,3,4-oxadiazol-2-amines (4a,b)

To the 10 ml methanolic solution of substituted benzohydrazides **3a,b** (5 mmol), cyanogen bromide (7.5 mmol) was added. The reaction mixture was refluxed on water bath for 5–7 h. The resulting solution was cooled and neutralized with sodium bicarbonate solution (5% w/v). The solid thus separated out was filtered, washed with water, dried and recrystallized from methanol.

#### 5-(2-Chlorophenyl)-1,3,4-oxadiazol-2-amine (**4a**)

Yield: 62%, mp: 165–168°C, lit. 164–166°C [32]. IR (KBr), v, cm^−1^: 742 (C-Cl), 1265, 1036 (C-O-C), 1649 (C=N), 3470, 3402 (NH_2_). ^1^H NMR (400 MHz, DMSO-*d_6_*, TMS): δ 7.35 (bs, 2H, NH_2_), 7.49 (t, *J* = 7.52 Hz, 1H, H-9), 7.55 (t, *J* = 7.36 Hz, 1H, H-10), 7.65 (d, *J* = 7.8 Hz, 1H, H-8), 7.70 (d, *J* = 7.48 Hz, 1H, H-11). ^13^C NMR (100 MHz, DMSO-*d_6_*, TMS): δ 127.53 (C-10), 128.12 (C-11), 128.69 (C-8), 129.81 (C-9), 131.43 (C-7), 135.18 (C-6), 157.53 (C-2), 163.50 (C-5). Anal. Calcd. for C_8_H_6_N_3_OCl: C, 49.12; H, 3.09; N, 21.48. Found: C, 49.25; H, 3.14; N, 21.41.

#### 5-(4-Chlorophenyl)-1,3,4-oxadiazol-2-amine (**4b**)

Yield: 70%, mp: 243–245°C, lit. 231–233°C [32]. IR (KBr), v, cm^−1^: 745 (C-Cl), 1277, 1043 (C-O-C), 1662 (C=N), 3480, 3396 (NH_2_). ^1^H NMR (400 MHz, DMSO-*d_6_*, TMS): δ 7.37 (bs, 2H, NH_2_), 7.62 (d, *J* = 8.24 Hz, 2H, H-8,10), 7.69 (d, *J* = 8.24 Hz, 2H, H-7,11). ^13^C NMR (100 MHz, DMSO-*d_6_*, TMS): δ 123.34 (C-6), 131.78 (C-7,11), 133.20 (8,10), 135.42 (C-9), 157.58 (C-2), 163.56 (C-5). Anal. Calcd. for C_8_H_6_N_3_OCl: C, 49.12; H, 3.09; N, 21.48. Found: C, 48.98; H, 3.17; N, 21.56.

### General procedure for 4-(5-substitutedphenyl-1,3,4-oxadiazol-2-yl)benzenamines (5a,b)

A mixture of 4-aminobenzoic acid (5 mmol) and substituted benzohydrazides **3a,b** (5 mmol) in 5 ml phosphorus oxychloride was refluxed on water bath for 7–10 h. After the completion of reaction, it was cooled and poured onto crushed ice with continuous stirring. The solid mass separated was neutralized with sodium bicarbonate solution (10% w/v). The resulting solid thus obtained was collected by filtration, washed well with cold water, dried and recrystallized from absolute ethanol.

#### 4-[5-(2-Chlorophenyl)-1,3,4-oxadiazol-2-yl]benzenamine (**5a**)

Yield: 63%, mp: 175–178°C. IR (KBr), v, cm^−1^: 746 (C-Cl), 1266, 1042 (C-O-C), 1653 (C=N), 3482, 3380 (NH_2_). ^1^H NMR (400 MHz, DMSO-*d_6_*, TMS): δ 5.46 (bs, 2H, NH_2_), 6.80 (d, *J* = 8.32 Hz, 2H, H-8,10), 7.31 (d, *J* = 8.32 Hz, 2H, H-7,11), 7.50 (t, *J* = 7.56 Hz, 1H, H-15), 7.56 (t, *J* = 7.4 Hz, 1H, H-16), 7.64 (d, *J* = 7.84 Hz, 1H, H-14), 7.68 (d, *J* = 7.5 Hz, 1H, H-17). ^13^C NMR (100 MHz, DMSO-*d_6_*, TMS): δ 108.13 (C-6), 114.84 (C-8,10), 127.53 (C-16), 128.14 (C-17), 128.62 (C-7,11), 128.74 (C-14), 129.85 (C-15), 131.38 (C-13), 135.22 (C-12), 148.48 (C-9), 162.37 (C-2,5). Anal. Calcd. for C_14_H_10_N_3_OCl: C, 61.89; H, 3.71; N, 15.47. Found: C, 61.76; H, 3.75; N, 15.39.

#### 4-[5-(4-Chlorophenyl)-1,3,4-oxadiazol-2-yl]benzenamine (**5b**)

Yield: 65%, mp: 210–214°C, lit. 210–212°C [12]. IR (KBr), v, cm^−1^: 743 (C-Cl), 1268, 1040 (C-O-C), 1658 (C=N), 3482, 3375 (NH_2_). ^1^H NMR (400 MHz, DMSO-*d_6_*, TMS): δ 5.45 (bs, 2H, NH_2_), 6.79 (d, *J* = 8.36 Hz, 2H, H-8,10), 7.29 (d, *J* = 8.36 Hz, 2H, H-7,11), 7.63 (d, *J* = 8.2 Hz, 2H, H-14,16), 7.68 (d, *J* = 8.2 Hz, 2H, H-13,17). ^13^C NMR (100 MHz, DMSO-*d_6_*, TMS): δ 107.42 (C-6), 114.45 (C-8,10), 123.31 (C-12), 128.73 (C-7,11), 131.82 (C-13,17), 133.24 (C-14,16), 135.44 (C-15), 148.53 (C-9), 161.89 (C-2,5). Anal. Calcd. for C_14_H_10_N_3_OCl: C, 61.89; H, 3.71; N, 15.47. Found: C, 61.78; H, 3.66; N, 15.54.

### General procedure for benzoxazinones (8a–c)

A mixture of {2-[(2,6-Dichlorophenyl)amino]phenyl}acetyl chloride (**6**) (10 mmol) and substituted anthranilic acids **7a–c** (10 mmol) in 20 ml pyridine were stirred at 0–5°C for 1 h, further stirred for 1 h at room temperature. After completion of reaction, a pasty mass obtained, was washed thoroughly with sodium bicarbonate (5 % w/v) to remove unreacted acid. A solid separated was filtered, dried and recrystallized from methanol.

#### 2-[2-(2,6-Dichlorophenyl)amino]benzyl-4*H*-3,1-benzoxazin-4-one (**8aF**

Yield: 53%, mp: 183–186°C, lit. 185–187 °C [33]. IR (KBr), v, cm^−1^: 745 (C-Cl), 1151 (CO), 1316 (C-N), 1620 (C=N), 1742 (C=O), 2925, 2851 (CH_2_), 3449 (NH). ^1^H NMR (400 MHz, DMSO-*d_6_*, TMS): δ 3.52 (s, 2H, H-11), 6.39 (d, *J* = 7.96 Hz, 1H, H-14), 6.88 (t, *J* = 7.4 Hz, 1H, H-16), 7.04–7.09 (m, 2H, H-15,22), 7.21 (d, *J* = 7.54 Hz, 1H, H-17), 7.42 (d, *J* = 8.08 Hz, 2H, H-21,23), 7.51 (d, *J* = 8.12 Hz, 1H, H-8), 7.84 (t, *J* = 7.8 Hz, 1H, H-7), 8.06 (t, *J* = 7.64 Hz, 1H, H-6), 8.12 (d, *J* = 7.72 Hz, 1H, H-5), 9.12 (bs, 1H, H-18). ^13^C NMR (100 MHz, DMSO-*d_6_*, TMS): δ 32.47 (C-11), 116.27 (C-16), 116.54 (C-10), 120.54 (C-14), 122.35 (C-8), 124.15 (C-22), 126.61 (C-15), 127.12 (C-12), 127.32 (C-21,23), 127.54 (C-6), 129.34 (C-20,24), 131.23 (C-17), 131.52 (C-5), 135.43 (C-7), 137.23 (C-19), 141.76 (C-13), 149.53 (C-9), 159.36 (C-4), 164.51 (C-2). Anal. Calcd. for C_21_H_14_Cl_2_N_2_O_2_: C, 63.49; H, 3.55; N, 7.05. Found: C, 63.45; H, 3.56; N, 7.03.

#### 6-Bromo-2-[2-(2,6-dichlorophenyl)amino]benzyl-4*H*-3,1-benzoxazin-4-one (**8b**)

Yield: 55%, mp: 194–198°C, lit. 193–196 °C. [34]. IR (KBr), v, cm^−1^: 565 (C-Br), 743 (C-Cl), 1153 (C-O), 1318 (C-N), 1618 (C=N), 1740 (C=O), 2926, 2850 (CH_2_), 3446 (NH). ^1^H NMR (400 MHz, DMSO-*d_6_*, TMS): δ 3.53 (s, 2H, H-11), 6.40 (d, *J* = 8 Hz, 1H, H-14), 6.88 (t, *J* = 7.44 Hz, 1H, H-16), 7.03–7.08 (m, 2H, H-15,22), 7.22 (d, *J* = 7.58 Hz, 1H, H-17), 7.41 (d, *J* = 8.16 Hz, 2H, H-21,23), 7.65 (d, *J* = 8.32 Hz, 1H, H-8), 8.12 (d, *J* = 8.32 Hz, 1H, H-7), 8.16 (s, 1H, H-5), 9.10 (bs, 1H, H-18). ^13^C NMR (100 MHz, DMSO-*d_6_*, TMS): δ 32.43 (C-11), 116.31 (C-16), 118.64 (C-10), 120.62 (C-14), 121.67 (C-6), 124.31 (C-22), 124.57 (C-8), 126.54 (C-15), 127.17 (C-12), 127.43 (C-21,23), 129.41 (C-20,24), 131.12 (C-17), 135.22 (C-5), 137.29 (C-19), 138.23 (C-7), 141.78 (C-13), 148.73 (C-9), 159.23 (C-4), 164.33 (C-2). Anal. Calcd. for C_21_H_13_BrCl_2_N_2_O_2_: C, 52.97; H, 2.75; N, 5.88. Found: C, 52.94; H, 2.74; N, 5.90.

#### 2-[2-(2,6-Dichlorophenyl)amino]benzyl-6-iodo-4H-3,1-benzoxazin-4-one (**8c**)

Yield: 58%, mp: 189–193°C. IR (KBr), v, cm^−1^: 620 (C-I), 747 (C-Cl), 1148 (C-O), 1320 (C-N), 1617 (C=N), 1745 (C=O), 2923, 2848 (CH_2_), 3450 (NH). ^1^H NMR (400 MHz, DMSO-*d_6_*, TMS): δ 3.53 (s, 2H, H-11), 6.41 (d, *J* = 7.92 Hz, 1H, H-14), 6.89 (t, *J* = 7.36 Hz, 1H, H-16), 7.04-7.09 (m, 2H, H-15,22), 7.22 (d, *J* = 7.54 Hz, 1H, H-17), 7.25 (d, *J* = 8.28 Hz, 1H, H-8), 7.42 (d, *J* = 8.12 Hz, 2H, H-21,23), 8.05 (d, *J* = 8.28 Hz, 1H, H-7), 8.48 (s, 1H, H-5), 9.10 (bs, 1H, H-18). ^13^C NMR (100 MHz, DMSO-*d_6_*, TMS): δ 32.53 (C-11), 93.14 (C-6), 116.25 (C-16), 118.23 (C-10), 120.57 (C-14), 123.74 (C-8), 124.19 (C-22), 126.58 (C-15), 127.05 (C-12), 127.33 (C-21,23), 129.39 (C-20,24), 131.14 (C-17), 137.42 (C-19), 138.87 (C-5), 141.81 (C-13), 144.27 (C-7), 148.62 (C-9), 159.53 (C-4), 164.47 (C-2). Anal. Calcd. for C_21_H_13_Cl_2_IN_2_O_2_: C, 48.21; H, 2.50; N, 5.35. Found: C, 48.25; H, 2.49; N, 5.33.

### General procedure for 1,3,4-oxadiazolyl-quinazolin-4(3H)-ones (9a–f)

A mixture of benzoxazinones **8a–c** (2.5 mmol) and 5-substituted phenyl-1,3,4-oxadiazol-2-amines **4a,b** (2.5 mmol) in 10 ml glacial acetic acid was refluxed under anhydrous condition for 4–6 h. After cooling it was poured into crushed ice. The solid separated out was filtered, thoroughly washed with cold distilled water, dried, and recrystallized from ethanol.

#### 3-[5-(2-Chlorophenyl)-1,3,4-oxadiazol-2-yl]-2-{2-[(2,6-dichlorophenyl)amino]benzyl}–quinazolin-4(3*H*)-one (**9a**)

Yield: 63%, mp: 233–235°C. IR (KBr), v, cm^−1^: 746 (C-Cl), 1318 (C-N), 1263, 1037 (C-O-C oxadiazole), 1609 (C=N quinazolinone), 1650 (C=N oxadiazole), 1678 (C=O quinazolinone), 2926, 2853 (CH_2_), 3445 (NH). ^1^H NMR (400 MHz, DMSO-*d_6_*, TMS): δ 3.52 (s, 2H, H-11), 6.41 (d, *J* = 7.96 Hz, 1H, H-14), 6.89 (t, *J* = 7.4 Hz, 1H, H-16), 7.03–7.09 (m, 2H, H-15,22), 7.22 (d, *J* = 7.54 Hz, 1H, H-17), 7.41 (d, *J* = 8.08 Hz, 2H, H-21,23), 7.47 (t, *J* = 7.68 Hz, 1H, H-6), 7.50 (t, *J* = 7.56 Hz, 1H, H-33), 7.57 (t, *J* = 7.4 Hz, 1H, H-34), 7.61 (d, *J* = 8.16 Hz, 1H, H-8), 7.67 (d, *J* = 7.84 Hz, 1H, H-32), 7.71 (d, *J* = 7.52 Hz, 1H, H-35), 7.76 (t, *J* = 7.84 Hz, 1H, H-7), 8.10 (d, *J* = 7.76 Hz, 1H, H-5), 9.09 (bs, 1H, H-18). ^13^C NMR (100 MHz, DMSO-*d_6_*, TMS): δ 32.54 (C-11), 116.32 (C-16), 120.41 (C-14), 120.77 (C-10), 122.54 (C-8), 124.31 (C-22), 126.54 (C-15), 127.21 (C-12), 127.39 (C-21,23), 127.49 (C-34), 127.58 (C-6), 128.12 (C-35), 128.55 (C-32), 128.76 (C-5), 129.35 (C-20,24), 129.81 (C-33), 131.17 (C-17), 131.45 (C-31), 133.66 (C-7), 135.24 (C-30), 137.32 (C-19), 141.68 (C-13), 147.15 (C-9), 156.72 (C-26), 160.74 (C-4), 163.27 (C-2), 164.65 (C-29). Anal. Calcd. for C_29_H_18_Cl_3_N_5_O_2_: C, 60.59; H, 3.16; N, 12.18. Found: C, 60.54; H, 3.11; N, 12.21.

#### 6-Bromo-3-[5-(2-chlorophenyl)-1,3,4-oxadiazol-2-yl]-2-{2-[(2,6-dichlorophenyl)amino]–benzyl}quinazolin-4(3H)-one (**9b**)

Yield: 55%, mp: 225–228°C. IR (KBr), v, cm^−1^: 570 (C-Br), 745 (C-Cl), 1311 (C-N), 1260, 1035 (C-O-C oxadiazole), 1611 (C=N quinazolinone), 1653 (C=N oxadiazole), 1675 (C=O quinazolinone), 2925, 2851 (CH_2_), 3447 (NH). ^1^H NMR (400 MHz, DMSO-*d_6_*, TMS): δ 3.53 (s, 2H, H-11), 6.39 (d, *J* = 8 Hz, 1H, H-14), 6.89 (t, *J* = 7.44 Hz, 1H, H-16), 7.03–7.09 (m, 2H, H-15,22), 7.21 (d, *J* = 7.58 Hz, 1H, H-17), 7.42 (d, *J* = 8.16 Hz, 2H, H-21,23), 7.51 (t, *J* = 7.52 Hz, 1H, H-33), 7.55 (t, *J* = 7.36 Hz, 1H, H-34), 7.61 (d, *J* = 8.24 Hz, 1H, H-8), 7.66 (d, *J* = 7.8 Hz, 1H, H-32), 7.70 (d, *J* = 7.48 Hz, 1H, H-35), 8.08 (d, *J* = 8.24 Hz, 1H, H-7), 8.12 (s, 1H, H-5), 9.08 (bs, 1H, H-18). ^13^C NMR (100 MHz, DMSO-*d_6_*, TMS): δ 32.65 (C-11), 116.24 (C-16), 120.53 (C-14), 123.13 (C-10), 124.24 (C-22), 124.58 (C-8), 126.47 (C-15), 127.18 (C-12), 127.37 (C-21,23), 127.48 (C-34), 127.66 (C-6), 128.15 (C-35), 128.72 (C-32), 129.34 (C-20,24), 129.79 (C-33), 131.16 (C-17), 131.47 (C-31), 132.25 (C-5), 135.21 (C-30), 136.37 (C-7), 137.36 (C-19), 141.77 (C-13), 146.21 (C-9), 156.64 (C-26), 160.59 (C-4), 163.38 (C-2), 164.57 (C-29). Anal. Calcd. for C_29_H_17_BrCl_3_N_5_O_2_: C, 53.28; H, 2.62; N, 10.71. Found: C, 53.19; H, 2.65; N, 10.75.

#### 3-[5-(2-Chlorophenyl)-1,3,4-oxadiazol-2-yl]-2-{2-[(2,6-dichlorophenyl)amino]benzyl}-6-iodoquinazolin-4(3*H*)-one (**9c**)

Yield: 66%, mp: 241–244°C. IR (KBr), v, cm^−1^: 618 (C-I), 740 (C-Cl), 1317 (C-N), 1262, 1042 (C-O-C oxadiazole), 1608 (C=N quinazolinone), 1655 (C=N oxadiazole), 1684 (C=O quinazolinone), 2928, 2854 (CH_2_), 3449 (NH). ^1^H NMR (400 MHz, DMSO-*d_6_*, TMS): δ 3.52 (s, 2H, H-11), 6.40 (d, *J* = 7.92 Hz, 1H, H-14), 6.89 (t, *J* = 7.36 Hz, 1H, H-16), 7.04–7.10 (m, 2H, H-15,22), 7.21 (d, *J* = 7.5 Hz, 1H, H-17), 7.25 (d, *J* = 8.28 Hz, 1H, H-8), 7.41 (d, *J* = 8.12 Hz, 2H, H-21,23), 7.48 (t, *J* = 7.56 Hz, 1H, H-33), 7.55 (t, *J* = 7.9 Hz, 1H, H-36), 7.64 (d, *J* = 7.84 Hz, 1H, H-32), 7.69 (d, *J* = 7.48 Hz, 1H, H-35), 7.93 (d, *J* = 8.28 Hz, 1H, H-7), 8.28 (s, 1H, H-5), 9.11 (bs, 1H, H-18). ^13^C NMR (100 MHz, DMSO-*d_6_*, TMS): δ 32.95 (C-11), 93.15 (C-6), 116.25 (C-16), 120.44 (C-14), 122.56 (C-10), 124.02 (C-8), 124.23 (C-22), 126.50 (C-15), 127.13 (C-12), 127.34 (C-21,23), 127.57 (C-34), 128.14 (C-35), 128.71 (C-32), 129.47 (C-20,24), 129.82 (C-33), 131.30 (C-17), 131.46 (C-31), 135.19 (C-30), 136.24 (C-5), 137.35 (C-19), 141.78 (C-13), 142.43 (C-7), 146.11 (C-9), 156.69 (C-26), 160.88 (C-4), 163.35 (C-2), 164.61 (C-29). Anal. Calcd. for C_29_H_17_Cl_3_IN_5_O_2_: C, 49.71; H, 2.45; N, 9.99. Found: C, 49.66; H, 2.51; N, 10.02.

#### 3-[5-(4-Chlorophenyl)-1,3,4-oxadiazol-2-yl]-2-{2-[(2,6-dichlorophenyl)amino]benzyl}–quinazolin-4(3*H*)-one (**9d**)

Yield: 65%, mp: 260–265°C. IR (KBr), v, cm^−1^: 748 (C-Cl), 1315 (C-N), 1264, 1038 (C-O-C oxadiazole), 1612 (C=N quinazolinone), 1648 (C=N oxadiazole), 1675 (C=O quinazolinone), 2923, 2848 (CH_2_), 3442 (NH). ^1^H NMR (400 MHz, DMSO-*d_6_*, TMS): δ 3.51 (s, 2H, H-11), 6.38 (d, *J* = 8.04 Hz, 1H, H-14), 6.89 (t, *J* = 7.48 Hz, 1H, H-16), 7.03–7.10 (m, 2H, H-15,22), 7.22 (d, *J* = 7.62 Hz, 1H, H-17), 7.43 (d, *J* = 8.12 Hz, 2H, H-21,23), 7.49 (t, *J* = 7.6 Hz, 1H, H-6), 7.61 (d, *J* = 8.08 Hz, 1H, H-8), 7.65 (d, *J* = 8.24 Hz, 2H, H-32,34), 7.70 (d, *J* = 8.24 Hz, 2H, H-31,35), 7.74 (t, *J* = 7.8 Hz, 1H, H-7), 8.11 (d, *J* = 7.72 Hz, 1H, H-5), 9.07 (bs, 1H, H-18). ^13^C NMR (100 MHz, DMSO-*d_6_*, TMS): δ 32.51 (C-11), 116.30 (C-16), 120.43 (C-14), 120.75 (C-10), 122.52 (C-8), 123.32 (C-30), 124.33 (C-22), 126.57 (C-15), 127.23 (C-12), 127.41 (C-21,23), 127.60 (C-6), 128.78 (C-5), 129.37 (C-20,24), 131.20 (C-17), 131.82 (C-31,35), 133.21 (C-32,34), 133.68 (C-7), 135.43 (C-33), 137.36 (C-19), 141.70 (C-13), 147.17 (C-9), 156.62 (C-26), 160.78 (C-4), 163.37 (C-2), 164.67 (C-29). Anal. Calcd. for C_29_H_18_Cl_3_N_5_O_2_: C, 60.59; H, 3.16; N, 12.18. Found: C, 60.51; H, 3.19; N, 12.15.

#### 6-Bromo-3-[5-(4-chlorophenyl)-1,3,4-oxadiazol-2-yl]-2-{2-[(2,6-dichlorophenyl)amino]–benzyl}quinazolin-4(3*H*)-one (**9e**)

Yield: 61%, mp: 254–258°C. IR (KBr), v, cm^−1^: 568 (C-Br), 743 (C-Cl), 1316 (C-N), 1258, 1032 (C-O-C oxadiazole), 1613 (C=N quinazolinone), 1650 (C=N oxadiazole), 1674 (C=O quinazolinone), 2922, 2849 (CH_2_), 3444 (NH). ^1^H NMR (400 MHz, DMSO-*d_6_*, TMS): δ 3.52 (s, 2H, H-11), 6.41 (d, *J* = 8 Hz, 1H, H-14), 6.90 (t, *J* = 7.44 Hz, 1H, H-16), 7.04–7.10 (m, 2H, H-15,22), 7.20 (d, *J* = 7.58 Hz, 1H, H-17), 7.42 (d, *J* = 8.08 Hz, 2H, H-21,23), 7.60 (d, *J* = 8.32 Hz, 1H, H-8), 7.64 (d, *J* = 8.2 Hz, 2H, H-32,34), 7.69 (d, *J* =8.2 Hz, 2H, H-31,35), 8.01 (d, *J* = 8.32 Hz, 1H, H-7), 8.09 (s, 1H, H-5), 9.08 (bs, 1H, H-18). ^13^C NMR (100 MHz, DMSO-*d_6_*, TMS): δ 32.57 (C-11), 116.28 (C-16), 120.56 (C-14), 123.16 (C-10), 123.33 (C-30), 124.27 (C-22), 124.56 (C-8), 126.48 (C-15), 127.22 (C-12), 127.39 (C-21,23), 127.67 (C-6), 129.35 (C-20,24), 131.18 (C-17), 131.79 (C-31,35), 132.24 (C-5), 133.24 (C-32,34), 135.39 (C-33), 136.35 (C-7), 137.38 (C-19), 141.76 (C-13), 146.23 (C-9), 156.58 (C-26), 160.61 (C-4), 163.41 (C-2), 164.54 (C-29). Anal. Calcd. for C_29_H_17_BrCl_3_N_5_O_2_: C, 53.28; H, 2.62; N, 10.71. Found: C, 53.35; H, 2.66; N, 10.58.

#### 3-[5-(4-Chlorophenyl)-1,3,4-oxadiazol-2-yl]-2-{2-[(2,6-dichlorophenyl)amino]benzyl}-6-iodoquinazolin-4(3*H*)-one (**9f**)

Yield: 68%, mp: 273–277°C. IR (KBr), v, cm^−1^: 615 (C-I), 746 (C-Cl), 1315 (C-N), 1260, 1045 (C-O-C oxadiazole), 1612 (C=N quinazolinone), 1651 (C=N oxadiazole), 1680 (C=O quinazolinone), 2927, 2852 (CH_2_), 3450 (NH). ^1^H NMR (400 MHz, DMSO-*d_6_*, TMS): δ 3.51 (s, 2H, H-11), 6.41 (d, *J* = 7.92 Hz, 1H, H-14), 6.88 (t, *J* = 7.36 Hz, 1H, H-16), 7.05–7.08 (m, 2H, H-15,22), 7.21 (d, *J* = 7.54 Hz, 1H, H-17), 7.25 (d, *J* = 8.36 Hz, 1H, H-8), 7.41 (d, *J* = 8.12 Hz, 2H, H-21,23), 7.62 (d, *J* = 8.28 Hz, 2H, H-32,34), 7.68 (d, *J* = 8.28 Hz, 2H, H-31,35), 7.95 (d, *J* = 8.36 Hz, 1H, H-7), 8.28 (s, 1H, H-5), 9.11 (bs, 1H, H-18). ^13^C NMR (100 MHz, DMSO-*d_6_*, TMS): δ 33.05 (C-11), 93.18 (C-6), 116.29 (C-16), 120.42 (C-14), 122.58 (C-10), 123.30 (C-30), 124.08 (C-8), 124.25 (C-22), 126.52 (C-15), 127.16 (C-12), 127.35 (C-21,23), 129.46 (C-20,24), 131.32 (C-17), 131.77 (C-31,35), 133.25 (C-32,34), 135.41 (C-33), 136.23 (C-5), 137.34 (C-19), 141.76 (C-13), 142.45 (C-7), 146.13 (C-9), 156.65 (C-26), 160.89 (C-4), 163.37 (C-2), 164.62 (C-29). Anal. Calcd. for C_29_H_17_Cl_3_IN_5_O_2_: C, 49.71; H, 2.45; N, 9.99. Found: C, 49.65; H, 2.47; N, 10.04.

### General procedure for 1,3,4-oxadiazolyl-quinazolin-4(3H)-ones (10a–f)

A mixture of benzoxazinones **8a–c** (2.5 mmol) and 4-(5-substituted phenyl-1,3,4-oxadiazol-2-yl)benzenamines **5a,b** (2.5 mmol) 10 ml in pyridine was refluxed on an oil bath for 6–8 h. After completion of the reaction, the oily mass was slowly poured onto crushed ice cold water containing 5 ml concentrated HCl with continues stirring. The product obtained was filtered and washed several times with cold water, dried and recrystallized from ethanol.

#### 3-{4-[5-(2-Chlorophenyl)-1,3,4-oxadiazol-2-yl]phenyl}-2-{2-[(2,6-dichlorophenyl)amino]–benzyl}quinazolin-4(3*H*)-one (**10a**)

Yield: 62%, mp: 228–231°C. IR (KBr), v, cm^−1^: 740 (C-Cl), 1316 (C-N), 1265, 1041 (C-O-C oxadiazole), 1610 (C=N quinazolinone), 1656 (C=N oxadiazole), 1676 (C=O quinazolinone), 2925, 2852 (CH_2_), 3446 (NH). ^1^H NMR (400 MHz, DMSO-*d_6_*, TMS): δ 3.51 (s, 2H, H-11), 6.40 (d, *J* = 8 Hz, 1H, H-14), 6.89 (t, *J* = 7.4 Hz, 1H, H-16), 7.03–7.09 (m, 2H, H-15,22), 7.22 (d, *J* = 7.58 Hz, 1H, H-17), 7.42 (d, *J* = 8.16 Hz, 2H, H-21,23), 7.45 (d, *J* = 8.4 Hz, 2H, H-26,30), 7.47–7.55 (m, 4H, Ar-H), 7.57 (t, *J* = 7.36 Hz, 1H, H-40), 7.62 (d, *J* = 8.16 Hz, 1H, H-8), 7.66 (d, *J* = 7.8 Hz, 1H, H-38), 7.71 (d, *J* = 7.48 Hz, 1H, H-41), 7.75 (t, *J* = 7.84 Hz, 1H, H-7), 8.07 (d, *J* = 7.74 Hz, 1H, H-5), 9.11 (bs, 1H, H-18). ^13^C NMR (100 MHz, DMSO-*d_6_*, TMS): δ 32.48 (C-11), 116.27 (C-16), 120.43 (C-14), 120.81 (C-10), 121.47 (C-28), 121.82 (C-26,30), 122.58 (C-8), 124.34 (C-22), 126.57 (C-15), 127.23 (C-12), 127.40 (C-21,23), 127.51 (C-40), 127.61 (C-6), 127.72 (C-27,29), 128.14 (C-41), 128.67 (C-38), 128.78 (C-5), 129.33 (C-20,24), 129.78 (C-39), 131.18 (C-17), 131.43 (C-37), 132.64 (C-25), 133.62 (C-7), 135.22 (C-36), 137.34 (C-19), 141.67 (C-13), 147.12 (C-9), 160.73 (C-4), 163.26 (C-2), 164.64 (C-32, 35). Anal. Calcd. for C_35_H_22_Cl_3_N_5_O_2_: C, 64.58; H, 3.41; N, 10.76. Found: C, 64.51; H, 3.46; N, 10.71.

#### 6-Bromo-3-{4-[5-(2-chlorophenyl)-1,3,4-oxadiazol-2-yl]phenyl}-2-{2-[(2,6-dichlorophenyl)–amino]benzyl}quinazolin-4(3*H*)-one (**10b**)

Yield: 64%, mp: 213–217°C. IR (KBr), v, cm^−1^: 572 (C-Br), 743 (C-Cl), 1312 (C-N), 1268, 1048 (C-O-C oxadiazole), 1609 (C=N quinazolinone), 1653 (C=N oxadiazole), 1681 (C=O quinazolinone), 2921, 2847 (CH_2_), 3442 (NH). ^1^H NMR (400 MHz, DMSO-*d_6_*, TMS): δ 3.53 (s, 2H, H-11), 6.42 (d, *J* = 7.96 Hz, 1H, H-14), 6.91 (t, *J* = 7.44 Hz, 1H, H-16), 7.03–7.10 (m, 2H, H-15,22), 7.23 (d, *J* = 7.58 Hz, 1H, H-17), 7.42 (d, *J* = 8.12 Hz, 2H, H-21,23), 7.45 (d, *J* = 8.36 Hz, 2H, H-26,30), 7.48 (t, *J* = 7.56 Hz, 1H, H-39), 7.54 (t, *J* = 7.4 Hz, 1H, H-40), 7.57 (d, *J* = 8.36 Hz, 2H, H-27,29), 7.61 (d, *J* = 8.4 Hz, 1H, H-8), 7.65 (d, *J* = 7.8 Hz, 1H, H-38), 7.72 (d, *J* = 7.48 Hz, 1H, H-41), 8.05 (d, *J* = 8.4 Hz, 1H, H-7), 8.12 (s, 1H, H-5), 9.11 (bs, 1H, H-18). ^13^C NMR (100 MHz, DMSO-*d_6_*, TMS): δ 32.63 (C-11), 116.27 (C-16), 120.55 (C-14), 121.52 (C-28), 121.78 (C-26,30), 123.12 (C-10), 124.26 (C-22), 124.62 (C-8), 126.50 (C-15), 127.17 (C-12), 127.38 (C-21,23), 127.53 (C-40), 127.64 (C-6), 127.73 (C-27,29), 128.17 (C-41), 128.76 (C-38), 129.37 (C-20,24), 129.82 (C-39), 131.20 (C-17), 131.45 (C-37), 132.27 (C-5), 132.64 (C-25), 135.23 (C-36), 136.35 (C-7), 137.32 (C-19), 141.74 (C-13), 146.23 (C-9), 160.60 (C-4), 163.28 (C-2), 164.59 (C-32,35). Anal. Calcd. for C_35_H_21_BrCl_3_N_5_O_2_: C, 57.60; H, 2.90; N, 9.60. Found: C, 57.52; H, 2.94; N, 9.63.

#### 3-{4-[5-(2-Chlorophenyl)-1,3,4-oxadiazol-2-yl]phenyl}-2-{2-[(2,6-dichlorophenyl)amino]–benzyl}-6-iodoquinazolin-4(3*H*)-one (**10c**)

Yield: 60%, mp: 235–238°C. IR (KBr), v, cm^−1^: 620 (C-I), 748 (C-Cl), 1312 (C-N), 1270, 1051 (C-O-C oxadiazole), 1611 (C=N quinazolinone), 1649 (C=N oxadiazole), 1678 (C=O quinazolinone), 2928, 2855 (CH_2_), 3448 (NH). ^1^H NMR (400 MHz, DMSO-*d_6_*, TMS): δ 3.52 (s, 2H, H-11), 6.41 (d, *J* = 8 Hz, 1H, H-14), 6.88 (t, *J* = 7.44 Hz, 1H, H-16), 7.05–7.11 (m, 2H, H-15,22), 7.23 (d, *J* = 7.54 Hz, 1H, H-17), 7.26 (d, *J* = 8.32 Hz, 1H, H-8), 7.43 (d, *J* = 8.04 Hz, 2H, H-21,23), 7.46 (d, *J* = 8.44 Hz, 2H, H-26,30), 7.49 (t, *J* = 7.6 Hz, 1H, H-39), 7.53 (d, *J* = 8.44 Hz, 2H, H-27,29), 7.56 (t, *J* = 7.42 Hz, 1H, H-40), 7.63 (d, *J* = 7.88 Hz, 1H, H-38), 7.69 (d, *J* = 7.56 Hz, 1H, H-41), 7.93 (d, *J* = 8.32 Hz, 1H, H-7), 8.28 (s, 1H, H-5), 9.09 (bs, 1H, H-18). ^13^C NMR (100 MHz, DMSO-*d_6_*, TMS): δ 32.90 (C-11), 93.21 (C-6), 116.29 (C-16), 120.48 (C-14), 121.49 (C-28), 121.81 (C-26,30), 122.55 (C-10), 124.05 (C-8), 124.25 (C-22), 126.49 (C-15), 127.15 (C-12), 127.38 (C-21,23), 127.49 (C-40), 127.68 (C-27,29), 128.12 (C-41), 128.68 (C-38), 129.44 (C-20,24), 129.74 (C-39), 131.29 (C-17), 131.40 (C-37), 132.58 (C-25), 135.18 (C-36), 136.21 (C-5), 137.37 (C-19), 141.75 (C-13), 142.46 (C-7), 146.09 (C-9), 160.95 (C-4), 163.22 (C-2), 164.63 (C-32,35). Anal. Calcd. for C_35_H_21_Cl_3_IN_5_O_2_: C, 54.11; H, 2.72; N, 9.02. Found: C, 54.18; H, 2.76; N, 8.95.

#### 3-{4-[5-(4-Chlorophenyl)-1,3,4-oxadiazol-2-yl]phenyl}-2-{2-[(2,6-dichlorophenyl)amino]–benzyl}quinazolin-4(3*H*)-one (**10d**)

Yield: 59%, mp: 245–248°C. IR (KBr), v, cm^−1^: 748 (C-Cl), 1318 (C-N), 1275, 1045 (C-O-C oxadiazole), 1613 (C=N quinazolinone), 1658 (C=N oxadiazole), 1680 (C=O quinazolinone), 2925, 2852 (CH_2_), 3451 (NH). ^1^H NMR (400 MHz, DMSO-*d_6_*, TMS): δ 3.52 (s, 2H, H-11), 6.40 (d, *J* = 7.88 Hz, 1H, H-14), 6.89 (t, *J* = 7.36 Hz, 1H, H-16), 7.04–7.09 (m, 2H, H-15,22), 7.21 (d, *J* = 7.5 Hz, 1H, H-17), 7.42 (d, *J* = 8.08 Hz, 2H, H-21,23), 7.45 (d, *J* = 8.36 Hz, 2H, H-26,30), 7.49 (t, *J* = 7.6 Hz, 1H, H-6), 7.56 (d, *J* = 8.36 Hz, 2H, H-27,29), 7.61 (d, *J* = 8.12 Hz, 1H, H-8), 7.65 (d, *J* = 8.28 Hz, 2H, H-37,41), 7.68 (d, *J* = 8.28 Hz, 2H, H-38,40), 7.75 (t, *J* = 7.8 Hz, 1H, H-7), 8.09 (d, *J* = 7.68 Hz, 1H, H-5), 9.10 (bs, 1H, H-18). ^13^C NMR (100 MHz, DMSO-*d_6_*, TMS): δ 32.61 (C-11), 116.30 (C-16), 120.45 (C-14), 120.75 (C-10), 121.52 (C-28), 121.78 (C-26,30), 122.57 (C-8), 123.28 (C-36), 124.32 (C-22), 126.51 (C-15), 127.19 (C-12), 127.37 (C-21,23), 127.56 (C-6), 127.68 (C-27,29), 128.74 (C-5), 129.32 (C-20,24), 131.15 (C-17), 131.83 (C-37,41), 132.59 (C-25), 133.19 (C-38,40), 133.64 (C-7), 135.42 (C-39), 137.34 (C-19), 141.65 (C-13), 147.13 (C-9), 160.77 (C-4), 163.38 (C-2), 164.61 (C-32,35). Anal. Calcd. for C_35_H_22_Cl_3_N_5_O_2_: C, 64.58; H, 3.41; N, 10.76. Found: C, 64.53; H, 3.44; N, 10.73.

#### 6-Bromo-3-{4-[5-(4-chlorophenyl)-1,3,4-oxadiazol-2-yl]phenyl}-2-{2-[(2,6-dichlorophenyl)–amino]benzyl}quinazolin-4(3*H*)-one (**10e**)

Yield: 68%, mp: 221–224°C. IR (KBr), v, cm^−1^: 565 (C-Br), 747 (C-Cl), 1317 (C-N), 1266, 1044 (C-O-C oxadiazole), 1609 (C=N quinazolinone), 1650 (C=N oxadiazole), 1676 (C=O quinazolinone), 2923, 2852 (CH_2_), 3446 (NH). ^1^H NMR (400 MHz, DMSO-*d_6_*, TMS): δ 3.53 (s, 2H, H-11), 6.41 (d, *J* = 7.96 Hz, 1H, H-14), 6.90 (t, *J* = 7.4 Hz, 1H, H-16), 7.04–7.10 (m, 2H, H-15,22), 7.20 (d, *J* = 7.58 Hz, 1H, H-17), 7.42 (d, *J* = 8.12 Hz, 2H, H-21,23), 7.46 (d, *J* = 8.4 Hz, 2H, H-26,30), 7.56 (d, *J* = 8.4 Hz, 2H, H-27,29), 7.60 (d, *J* = 8.28 Hz, 1H, H-8), 7.64 (d, *J* = 8.24 Hz, 2H, H-38,40), 7.69 (d, *J* = 8.24 Hz, 2H, H-37,41), 8.07 (d, *J* = 8.28 Hz, 1H, H-7), 8.13 (s, 1H, H-5), 9.11 (bs, 1H, H-18). ^13^C NMR (100 MHz, DMSO-*d_6_*, TMS): δ 32.67 (C-11), 116.25 (C-16), 120.56 (C-14), 121.53 (C-28), 121.79 (C-26,30), 123.16 (C-10), 123.32 (C-36), 124.27 (C-22), 124.56 (C-8), 126.44 (C-15), 127.15 (C-12), 127.34 (C-21,23), 127.62 (C-6), 127.74 (C-27,29), 129.38 (C-20,24), 131.22 (C-17), 131.82 (C-37,41), 132.28 (C-5), 132.62 (C-25), 133.22 (C-38,40), 135.41 (C-39), 136.35 (C-7), 137.32 (C-19), 141.73 (C-13), 146.25 (C-9), 160.56 (C-4), 163.37 (C-2), 164.52 (C-32,35). Anal. Calcd. for C_35_H_21_BrCl_3_N_5_O_2_: C, 57.60; H, 2.90; N, 9.60. Found: C, 57.53; H, 2.93; N, 9.55.

#### 3-{4-[5-(4-Chlorophenyl)-1,3,4-oxadiazol-2-yl]phenyl}-2-{2-[(2,6-dichlorophenyl)amino]–benzyl}-6-iodoquinazolin-4(3*H*)-one (**10f**)

Yield: 65%, mp: 257–261°C. IR (KBr), v, cm^−1^: 617 (C-I), 745 (C-Cl), 1314 (C-N), 1268, 1052 (C-O-C oxadiazole), 1608 (C=N quinazolinone), 1652 (C=N oxadiazole), 1679 (C=O quinazolinone), 2925, 2851 (CH_2_), 3443 (NH). ^1^H NMR (400 MHz, DMSO-*d_6_*, TMS): δ 3.51 (s, 2H, H-11), 6.39 (d, *J* = 8 Hz, 1H, H-14), 6.88 (t, *J* = 7.48 Hz, 1H, H-16), 7.03–7.10 (m, 2H, H-15,22), 7.20 (d, *J* = 7.58 Hz, 1H, H-17), 7.24 (d, *J* = 8.24 Hz, 1H, H-8), 7.43 (d, *J* = 8.16 Hz, 2H, H-21,23), 7.47 (d, *J* = 8.32 Hz, 2H, H-26,30), 7.58 (d, *J* = 8.32 Hz, 2H, H-27,29), 7.63 (d, *J* = 8.2 Hz, 2H, H-38,40), 7.68 (d, *J* = 8.2 Hz, 2H, H-37,41), 7.95 (d, *J* = 8.24 Hz, 1H, H-7), 8.28 (s, 1H, H-5), 9.12 (bs, 1H, H-18). ^13^C NMR (100 MHz, DMSO-*d_6_*, TMS): δ 33.01 (C-11), 93.20 (C-6), 116.21 (C-16), 120.43 (C-14), 121.48 (C-28), 121.76 (C-26,30), 122.55 (C-10), 123.34 (C-36), 124.06 (C-8), 124.25 (C-22), 126.48 (C-15), 127.14 (C-12), 127.32 (C-21,23), 127.67 (C-27,29), 129.43 (C-20,24), 131.33 (C-17), 131.82 (C-37,41), 132.63 (C-25), 133.23 (C-38,40), 135.44 (C-39), 136.28 (C-5), 137.34 (C-19), 141.76 (C-13), 142.48 (C-7), 146.15 (C-9), 160.93 (C-4), 163.31 (C-2), 164.59 (C-32,35). Anal. Calcd. for C_35_H_21_Cl_3_IN_5_O_2_: C, 54.11; H, 2.72; N, 9.02. Found: C, 54.06; H, 2.75; N, 9.09.

### General procedure for quinazolin-4(3H)-ones (11a–c)

A mixture of benzoxazinones **8a–c** (5 mmol) and glycine (5 mmol) was refluxed in 20 ml butanol for 6–8 h. After completion of the reaction, it was concentrated and after cooling, water was added and solid thus obtained was filtered off and recrystallized from ethanol.

#### [2-{2-[(2,6-Dichlorophenyl)amino]benzyl}-4-oxoquinazolin-3(4*H*)-yl]acetic acid (**11a**)

Yield: 53%, mp: 202–206°C. IR (KBr), v, cm^−1^: 741 (C-Cl), 1315 (C-N), 1608 (C=N quinazolinone), 1686 (C=O quinazolinone), 1715 (C=O), 2780 (OH), 2924, 2851 (CH_2_), 3447 (NH). ^1^H NMR (400 MHz, DMSO-*d_6_*, TMS): δ 3.52 (s, 2H, H-11), 4.15 (s, 2H, H-25), 6.39 (d, *J* = 7.96 Hz, 1H, H-14), 6.88 (t, *J* = 7.44 Hz, 1H, H-16), 7.04–7.09 (m, 2H, H-15,22), 7.21 (d, *J* = 7.58 Hz, 1H, H-17), 7.42 (d, *J* = 8.08 Hz, 2H, H-21,23), 7.48 (t, *J* = 7.72 Hz, 1H, H-6), 7.62 (d, *J* = 8.2 Hz, 1H, H-8), 7.75 (t, *J* = 7.88 Hz, 1H, H-7), 8.10 (d, *J* = 7.8 Hz, 1H, H-5), 9.10 (bs, 1H, H-18), 12.34 (bs, 1H, H-26). ^13^C NMR (100 MHz, DMSO-*d_6_*, TMS): δ 32.62 (C-11), 42.93 (C-25), 116.31 (C-16), 120.44 (C-14), 120.91 (C-10), 122.52 (C-8), 124.29 (C-22), 126.51 (C-15), 127.18 (C-12), 127.37 (C-21,23), 127.82 (C-6), 128.71 (C-5), 129.32 (C-20,24), 131.14 (C-17), 135.74 (C-7), 137.28 (C-19), 141.71 (C-13), 147.13 (C-9), 160.72 (C-4), 164.63 (C-2), 173.52 (C-26). Anal. Calcd. for C_23_H_17_Cl_2_N_3_O_3_: C, 60.81; H, 3.77; N, 9.25. Found: C, 60.75; H, 3.70; N, 9.28.

#### [6-Bromo-2-{2-[(2,6-dichlorophenyl)amino]benzyl}-4-oxoquinazolin-3(4*H*)-yl]acetic acid (**11b**)

Yield: 56%, mp: 223–227°C. IR (KBr), v, cm^−1^: 567 (C-Br), 744 (C-Cl), 1317 (C-N), 1610 (C=N quinazolinone), 1684 (C=O quinazolinone), 1718 (C=O), 2785 (OH), 2925, 2849 (CH_2_), 3445 (NH). ^1^H NMR (400 MHz, DMSO-*d_6_*, TMS): δ 3.53 (s, 2H, H-11), 4.14 (s, 2H, H-25), 6.40 (d, *J* = 8 Hz, 1H, H-14), 6.88 (t, *J* = 7.4 Hz, 1H, H-16), 7.03–7.08 (m, 2H, H-15,22), 7.22 (d, *J* = 7.5 Hz, 1H, H-17), 7.41 (d, *J* = 8.12 Hz, 2H, H-21,23), 7.63 (d, *J* = 8.36 Hz, 1H, H-8), 7.95 (d, *J* = 8.36 Hz, 1H, H-7), 8.03 (s, 1H, H-5), 9.09 (bs, 1H, H-18), 12.35 (bs, 1H, H-26). ^13^C NMR (100 MHz, DMSO-*d_6_*, TMS): δ 32.73 (C-11), 43.32 (C-25), 116.28 (C-16), 120.57 (C-14), 121.65 (C-6), 122.94 (C-10), 124.19 (C-22), 124.51 (C-8), 126.42 (C-15), 127.15 (C-12), 127.38 (C-21,23), 129.31 (C-20,24), 131.22 (C-17), 132.14 (C-5), 137.37 (C-19), 138.33 (C-7), 141.81 (C-13), 145.83 (C-9), 160.93 (C-4), 164.55 (C-2), 173.47 (C-26). Anal. Calcd. for C_23_H_16_BrCl_2_N_3_O_3_: C, 51.81; H, 3.02; N, 7.88. Found: C, 51.85; H, 3.05; N, 7.84.

#### [2-{2-[(2,6-Dichlorophenyl)amino]benzyl}-6-iodo-4-oxoquinazolin-3(4*H*)-yl]acetic acid (**11c**)

Yield: 60%, mp: 211–214°C. IR (KBr), v, cm^−1^: 618 (C-I), 746 (C-Cl), 1318 (C-N), 1612 (C=N quinazolinone), 1682 (C=O quinazolinone), 1713 (C=O), 2778 (OH), 2922, 2846 (CH_2_), 3443 (NH). ^1^H NMR (400 MHz, DMSO-*d_6_*, TMS): δ 3.53 (s, 2H, H-11), 4.16 (s, 2H, H-25), 6.41 (d, *J* = 7.92 Hz, 1H, H-14), 6.89 (t, *J* = 7.36 Hz, 1H, H-16), 7.04–7.09 (m, 2H, H-15,22), 7.22 (d, *J* = 7.5 Hz, 1H, H-17), 7.26 (d, *J* = 8.32 Hz, 1H, H-8), 7.42 (d, *J* = 8.04 Hz, 2H, H-21,23), 7.94 (d, *J* = 8.32 Hz, 1H, H-7), 8.27 (s, 1H, H-5), 9.09 (bs, 1H, H-18), 12.35 (bs, 1H, H-26). ^13^C NMR (100 MHz, DMSO-*d_6_*, TMS): δ 33.12 (C-11), 43.26 (C-25), 93.32 (C-6), 116.26 (C-16), 120.51 (C-14), 122.52 (C-10), 123.92 (C-8), 124.13 (C-22), 126.58 (C-15), 126.98 (C-12), 127.26 (C-21,23), 129.32 (C-20,24), 131.24 (C-17), 136.14 (C-5), 137.28 (C-19), 141.67 (C-13), 142.45 (C-7), 145.74 (C-9), 161.16 (C-4), 164.62 (C-2), 173.44 (C-26). Anal. Calcd. for C_23_H_16_Cl_2_IN_3_O_3_: C, 47.61; H, 2.78; N, 7.24. Found: C, 47.57; H, 2.75; N, 7.25.

### General procedure for 1,3,4-oxadiazolyl-quinazolin-4(3H)-ones (12a–f)

A mixture of **11a–c** (2.5 mmol), benzohydrazides **3a,b** (2.5 mmol) and 7 ml phosphorus trichloride in 10 ml dry benzene was refluxed under anhydrous condition for 10–12 h. After completion of reaction, benzene was distilled off under reduced pressure and the residue poured on to crushed ice and neutralized with sodium bicarbonate (5% w/v). The solid thus obtained was filtered, washed with cold water and recrystallized from ethanol.

#### 3-{[5-(2-Chlorophenyl)-1,3,4-oxadiazol-2-yl]methyl}-2-{2-[(2,6-dichlorophenyl)amino]-benzyl}quinazolin-4(3*H*)-one (**12a**)

Yield: 72%, mp: 181–184°C. IR (KBr), v, cm^−1^: 744 (C-Cl), 1313 (C-N), 1259, 1035 (C-O-C oxadiazole), 1611 (C=N quinazolinone), 1653 (C=N oxadiazole), 1678 (C=O quinazolinone), 2923, 2855 (CH_2_), 3448 (NH). ^1^H NMR (400 MHz, DMSO-*d_6_*, TMS): δ 3.51 (s, 2H, H-11), 4.49 (s, 2H, H-25), 6.40 (d, *J* = 7.96 Hz, 1H, H-14), 6.89 (t, *J* = 7.44 Hz, 1H, H-16), 7.05–7.10 (m, 2H, H-15,22), 7.22 (d, *J* = 7.58 Hz, 1H, H-17), 7.42 (d, *J* = 8.12 Hz, 2H, H-21,23), 7.47 (t, *J* = 7.56 Hz, 1H, H-34), 7.51 (t, *J* = 7.64 Hz, 1H, H-6), 7.56 (t, *J* = 7.4 Hz, 1H, H-35), 7.61 (d, *J* = 8.12 Hz, 1H, H-8), 7.67 (d, *J* = 7.84 Hz, 1H, H-33), 7.71 (d, *J* = 7.48 Hz, 1H, H-36), 7.75 (t, *J* = 7.8 Hz, 1H, H-7), 8.09 (d, *J* = 7.72 Hz, 1H, H-5), 9.10 (bs, 1H, H-18). ^13^C NMR (100 MHz, DMSO-*d_6_*, TMS): δ 33.02 (C-11), 42.42 (C-25), 116.32 (C-16), 120.45 (C-14), 120.93 (C-10), 122.57 (C-8), 124.31 (C-22), 126.54 (C-15), 127.22 (C-12), 127.39 (C-21,23), 127.61 (C-35), 127.83 (C-6), 128.24 (C-36), 128.73 (C-5), 128.82 (C-33), 129.37 (C-20,24), 129.75 (C-34), 131.18 (C-17), 131.79 (C-32), 135.47 (C-31), 135.81 (C-7), 137.34 (C-19), 141.75 (C-13), 147.18 (C-9), 156.28 (C-27), 160.74 (C-4), 163.46 (C-2), 164.65 (C-30). Anal. Calcd. for C_30_H_20_Cl_3_N_5_O_2_: C, 61.19; H, 3.42; N, 11.89. Found: C, 61.12; H, 3.47; N, 11.85.

#### 6-Bromo-3-{[5-(2-chlorophenyl)-1,3,4-oxadiazol-2-yl]methyl}-2-{2-[(2,6-dichlorophenyl)-amino]benzyl}quinazolin-4(3*H*)-one (**12b**)

Yield: 68%, mp: 175–178°C. IR (KBr), v, cm^−1^: 568 (C-Br), 742 (C-Cl), 1311 (C-N), 1263, 1045 (C-O-C oxadiazole), 1612 (C=N quinazolinone), 1653 (C=N oxadiazole), 1675 (C=O quinazolinone), 2922, 2850 (CH_2_), 3443 (NH). ^1^H NMR (400 MHz, DMSO-*d_6_*, TMS): δ 3.53 (s, 2H, H-11), 4.48 (s, 2H, H-25), 6.40 (d, *J* = 8 Hz, 1H, H-14), 6.88 (t, *J* = 7.48 Hz, 1H, H-16), 7.03–7.08 (m, 2H, H-15,22), 7.22 (d, *J* = 7.58 Hz, 1H, H-17), 7.41 (d, *J* = 8.08 Hz, 2H, H-21,23), 7.49 (t, *J* = 7.52 Hz, 1H, H-34), 7.56 (t, *J* = 7.36 Hz, 1H, H-35), 7.62 (d, *J* = 8.28 Hz, 1H, H-8), 7.67 (d, *J* = 7.8 Hz, 1H, H-33), 7.72 (d, *J* = 7.48 Hz, 1H, H-36), 8.06 (d, *J* = 8.28 Hz, 1H, H-7), 8.11 (s, 1H, H-5), 9.10 (bs, 1H, H-18). ^13^C NMR (100 MHz, DMSO-*d_6_*, TMS): δ 33.14 (C-11), 42.47 (C-25), 116.32 (C-16), 120.63 (C-14), 121.68 (C-6), 122.96 (C-10), 124.22 (C-22), 124.54 (C-8), 126.46 (C-15), 127.17 (C-12), 127.42 (C-21,23), 127.67 (C-35), 128.26 (C-36), 128.72 (C-33), 129.35 (C-20,24), 129.69 (C-34), 131.24 (C-17), 131.84 (C-32), 132.16 (C-5), 135.52 (C-31), 137.43 (C-19), 138.37 (C-7), 141.84 (C-13), 145.85 (C-9), 156.25 (C-27), 160.97 (C-4), 163.41 (C-2), 164.56 (C-30). Anal. Calcd. for C_30_H_19_BrCl_3_N_5_O_2_: C, 53.96; H, 2.87; N, 10.49. Found: C, 53.88; H, 2.85; N, 10.53.

#### 3-{[5-(2-Clorophenyl)-1,3,4-oxadiazol-2-yl]methyl}-2-{2-[(2,6-dichlorophenyl)amino]benzyl}-6-iodoquinazolin-4(3*H*)-one (**12c**)

Yield: 62%, mp: 196–98°C. IR (KBr), v, cm^−1^: 620 (C-I), 745 (C-Cl), 1316 (C-N), 1265, 1036 (C-O-C oxadiazole), 1609 (C=N quinazolinone), 1650 (C=N oxadiazole), 1678 (C=O quinazolinone), 2924, 2850 (CH_2_), 3445 (NH). ^1^H NMR (400 MHz, DMSO-*d_6_*, TMS): δ 3.53 (s, 2H, H-11), 4.48 (s, 2H, H-25), 6.41 (d, *J* = 8 Hz, 1H, H-14), 6.89 (t, *J* = 7.48 Hz, 1H, H-16), 7.04–7.09 (m, 2H, H-15,22), 7.22 (d, *J* = 7.62 Hz, 1H, H-17), 7.26 (d, *J* = 8.36 Hz, 1H, H-8), 7.42 (d, *J* = 8.12 Hz, 2H, H-21,23), 7.50 (t, *J* = 7.52 Hz, 1H, H-34), 7.56 (t, *J* = 7.36 Hz, 1H, H-35), 7.65 (d, *J* = 7.8 Hz, 1H, H-33), 7.71 (d, *J* = 7.44 Hz, 1H, H-36), 7.94 (d, *J* = 8.36 Hz, 1H, H-7), 8.27 (s, 1H, H-5), 9.09 (bs, 1H, H-18). ^13^C NMR (100 MHz, DMSO-*d_6_*, TMS): δ 33.51 (C-11), 42.47 (C-25), 93.33 (C-6), 116.27 (C-16), 120.53 (C-14), 122.55 (C-10), 123.91 (C-8), 124.16 (C-22), 126.62 (C-15), 127.11 (C-12), 127.28 (C-21,23), 127.64 (C-35), 128.26 (C-36), 128.73 (C-33), 129.37 (C-20,24), 129.75 (C-34), 131.28 (C-17), 131.84 (C-32), 136.15 (C-5), 135.52 (C-31), 137.31 (C-19), 141.70 (C-13), 142.48 (C-7), 145.77 (C-9), 156.23 (C-27), 161.18 (C-4), 163.44 (C-2), 164.67 (C-30). Anal. Calcd. for C_30_H_19_Cl_3_IN_5_O_2_: C, 50.41; H, 2.68; N, 9.80. Found: C, 50.45; H, 2.71; N, 9.73.

#### 3-{[5-(4Chlorophenyl)-1,3,4-oxadiazol-2-yl]methyl}-2-{2-[(2,6-dichlorophenyl)amino]-benzyl}quinazolin-4(3H)-one (**12d**)

Yield: 71%, mp: 227–30°C. IR (KBr), v, cm^−1^: 748 (C-Cl), 1316 (C-N), 1254, 1038 (C-O-C oxadiazole), 1608 (C=N quinazolinone), 1655 (C=N oxadiazole), 1683 (C=O quinazolinone), 2922, 2851 (CH_2_), 3443 (NH). ^1^H NMR (400 MHz, DMSO-*d_6_*, TMS): δ 3.52 (s, 2H, H-11), 4.47 (s, 2H, H-25), 6.39 (d, *J* = 8.04 Hz, 1H, H-14), 6.88 (t, *J* = 7.52 Hz, 1H, H-16), 7.04–7.09 (m, 2H, H-15,22), 7.21 (d, *J* = 7.62 Hz, 1H, H-17), 7.42 (d, *J* = 8.16 Hz, 2H, H-21,23), 7.50 (t, *J* = 7.68 Hz, 1H, H-6), 7.62 (d, *J* = 8.16 Hz, 1H, H-8), 7.65 (d, *J* = 8.2 Hz, 2H, H-33,35), 7.70 (d, *J* = 8.2 Hz, 2H, H-32,36), 7.74 (t, *J* = 7.84 Hz, 1H, H-7), 8.08 (d, *J* = 7.76 Hz, 1H, H-5), 9.12 (bs, 1H, H-18). ^13^C NMR (100 MHz, DMSO-*d_6_*, TMS): δ 33.05 (C-11), 42.23 (C-25), 116.35 (C-16), 120.48 (C-14), 120.96 (C-10), 122.55 (C-8), 123.34 (C-31), 124.34 (C-22), 126.53 (C-15), 127.26 (C-12), 127.34 (C-21,23), 127.77 (C-6), 128.74 (C-5), 129.35 (C-20,24), 131.21 (C-17), 131.83 (C-32,36), 133.26 (C-33,35), 135.38 (C-34), 135.78 (C-7), 137.36 (C-19), 141.76 (C-13), 147.18 (C-9), 156.27 (C-27), 160.78 (C-4), 163.38 (C-2), 164.67 (C-30). Anal. Calcd. for C_30_H_20_Cl_3_N_5_O_2_: C, 61.19; H, 3.42; N, 11.89. Found: C, 61.22; H, 3.38; N, 11.84.

#### 6-Bromo-3-{[5-(4chlorophenyl)-1,3,4-oxadiazol-2-yl]methyl}-2-{2-[(2,6-dichlorophenyl)-amino]benzyl}quinazolin-4(3*H*)-one (**12e**)

Yield: 67%, mp: 266–69°C. IR (KBr), v, cm^−1^: 571 (C-Br), 750 (C-Cl), 1313 (C-N), 1263, 1041 (C-O-C oxadiazole), 1607 (C=N quinazolinone), 1656 (C=N oxadiazole), 1678 (C=O quinazolinone), 2925, 2853 (CH_2_), 3448 (NH). ^1^H NMR (400 MHz, DMSO-*d_6_*, TMS): δ 3.53 (s, 2H, H-11), 4.49 (s, 2H, H-25), 6.40 (d, *J* = 7.88 Hz, 1H, H-14), 6.89 (t, *J* = 7.32 Hz, 1H, H-16), 7.02–7.08 (m, 2H, H-15,22), 7.20 (d, *J* = 7.46 Hz, 1H, H-17), 7.42 (d, *J* = 8.04 Hz, 2H, H-21,23), 7.60 (d, *J* = 8.32 Hz, 1H, H-8), 7.65 (d, *J* = 8.24 Hz, 2H, H-33,35), 7.72 (d, *J* = 8.24 Hz, 2H, H-32,36), 8.05 (d, *J* = 8.32 Hz, 1H, H-7), 8.12 (s, 1H, H-5), 9.08 (bs, 1H, H-18). ^13^C NMR (100 MHz, DMSO-*d_6_*, TMS): δ 33.15 (C-11), 42.51 (C-25), 116.25 (C-16), 120.54 (C-14), 121.63 (C-6), 122.87 (C-10), 123.30 (C-31), 124.15 (C-22), 124.46 (C-8), 126.38 (C-15), 127.17 (C-12), 127.42 (C-21,23), 129.28 (C-20,24), 131.25 (C-17), 131.78 (C-32,36), 132.12 (C-5), 133.23 (C-33,35), 135.46 (C-34), 137.42 (C-19), 138.32 (C-7), 141.83 (C-13), 145.85 (C-9), 156.31 (C-27), 160.94 (C-4), 163.37 (C-2), 164.57 (C-30). Anal. Calcd. for C_30_H_19_BrCl_3_N_5_O_2_: C, 53.96; H, 2.87; N, 10.49. Found: C, 53.91; H, 2.82; N, 10.48.

#### 3-{[5-(4Clorophenyl)-1,3,4-oxadiazol-2-yl]methyl}-2-{2-[(2,6-dichlorophenyl)amino]benzyl}-6-iodoquinazolin-4(3*H*)-one (**12f**)

Yield: 75%, mp: 243–46°C. IR (KBr), v, cm^−1^: 618 (C-I), 749 (C-Cl), 1315 (C-N), 1260, 1049 (C-O-C oxadiazole), 1611 (C=N quinazolinone), 1658 (C=N oxadiazole), 1683 (C=O quinazolinone), 2921, 2849 (CH_2_), 3445 (NH). ^1^H NMR (400 MHz, DMSO-*d_6_*, TMS): δ 3.51 (s, 2H, H-11), 4.48 (s, 2H, H-25), 6.42 (d, *J* = 7.96 Hz, 1H, H-14), 6.90 (t, *J* = 7.4 Hz, 1H, H-16), 7.03-7.08 (m, 2H, H-15,22), 7.21 (dd, *J* = 7.5 Hz, 1.28 Hz, 1H, H-17), 7.24 (d, *J* = 8.4 Hz, 1H, H-8), 7.41 (d, *J* = 8.12 Hz, 2H, H-21,23), 7.62 (d, *J* = 8.2 Hz, 2H, H-33,35), 7.68 (d, *J* = 8.2 Hz, 2H, H-32,36), 7.93 (d, *J* = 8.4 Hz, 1H, H-7), 8.28 (s, 1H, H-5), 9.12 (bs, 1H, H-18). ^13^C NMR (100 MHz, DMSO-*d_6_*, TMS): δ 33.47 (C-11), 42.45 (C-25), 93.38 (C-6), 116.31 (C-16), 120.56 (C-14), 122.54 (C-10), 123.33 (C-31), 123.97 (C-8), 124.17 (C-22), 126.60 (C-15), 127.02 (C-12), 127.25 (C-21,23), 129.36 (C-20,24), 131.27 (C-17), 131.77 (C-32,36), 133.29 (C-33,35), 135.45 (C-34), 136.18 (C-5), 137.33 (C-19), 141.71 (C-13), 142.48 (C-7), 145.76 (C-9), 156.31 (C-27), 161.14 (C-4), 163.49 (C-2), 164.63 (C-30). Anal. Calcd. for C_30_H_19_Cl_3_IN_5_O_2_: C, 50.41; H, 2.68; N, 9.80. Found: C, 50.35; H, 2.76; N, 9.77.

### Antimicrobial activity

The MICs of synthesized compounds were carried out by broth microdilution method as described by Rattan [35]. Antibacterial activity was screened against two gram positive bacteria (*S. aureus* MTCC 96, *S. pyogenes* MTCC 442) and two gram negative bacteria (*E. coli* MTCC 443, *P. aeruginosa* MTCC 1688). Ampicillin was used as a standard antibacterial agent. Antifungal activity was screened against three fungal species *C. albicans* MTCC 227, *A. niger* MTCC 282 and *A. clavatus* MTCC 1323. Griseofulvin was used as a standard antifungal agent.

All MTCC cultures were collected from Institute of Microbial Technology, Chandigarh and tested against above mentioned known drugs. Mueller hinton broth was used as nutrient medium to grow and dilute the drug suspension for the test. Inoculum size for test strain was adjust to 10^8^ CFU (Colony Forming Unit) per milliliter by comparing the turbidity. DMSO was used as diluents to get desired concentration of drugs to test upon standard bacterial strains. Serial dilutions were prepared in primary and secondary screening. The control tube containing no antibiotic was immediately sub cultured (before inoculation) by spreading a loopful evenly over a quarter of plate of medium suitable for the growth of the test organism and put for incubation at 37 °C overnight. The tubes were then incubated overnight. The MIC of the control organism was read to check the accuracy of the drug concentrations. The lowest concentration inhibiting growth of the organism was recorded as the MIC. All the tubes not showing visible growth (in the same manner as control tube described above) was sub cultured and incubated overnight at 37 °C. The amount of growth from the control tube before incubation (which represents the original inoculum) was compared. Subcultures might show: similar number of colonies indicating bacteriostatic; a reduced number of colonies indicating a partial or slow bactericidal activity and no growth if the whole inoculum has been killed. The test must include a second set of the same dilutions inoculated with an organism of known sensitivity. Each synthesized drug was diluted obtaining 2000 μg/ml concentration, as a stock solution. In primary screening 500 μg/ml, 250 μg/ml and 125 μg/ml concentrations of the synthesized drugs were taken. The active synthesized drugs found in this primary screening were further tested in a second set of dilution against all microorganisms. The drugs found active in primary screening were similarly diluted to obtain 100 μg/ml, 50 μg/ml, 25 μg/ml, 12.5 μg/ml, 6.250 μg/ml, 3.125 μg/ml and 1.5625 μg/ml concentrations. The highest dilution showing at least 99 % inhibition is taken as MIC.

## Conclusions

Aminosubstituted 1,3,4-oxadiazoles **4a,b** and **5a,b** exhibited very good antimicrobial activity. But when they were condensed with benzoxazinone formed oxadiazolyl-quinazolinone, showed increasing activity. Antimicrobial results were found uneven but most of the bromo and iodo derivatives of quinazolinone possessed very good antimicrobial activity. Furthermore CH_2_ link between 3^rd^ position of quinazolinone and 2^nd^ position of oxadiazole were found most active than other two series. So, it seems from the antimicrobial results that halogen atom and CH_2_ link played vital role in increasing antimicrobial activity.

## Figures and Tables

**Fig. 1.**
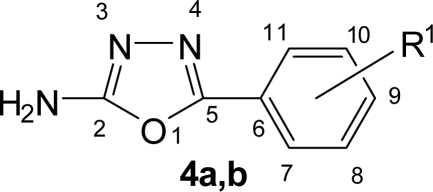
Numbering of 5-substituted phenyl-1,3,4-oxadiazol-2-amines **4a,b**

**Fig. 2.**
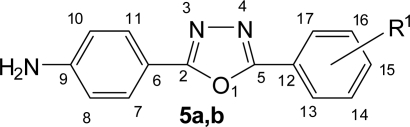
Numbering of 4-(5-substituted phenyl-1,3,4-oxadiazol-2-yl)benzenamines **5a,b**

**Fig. 3.**
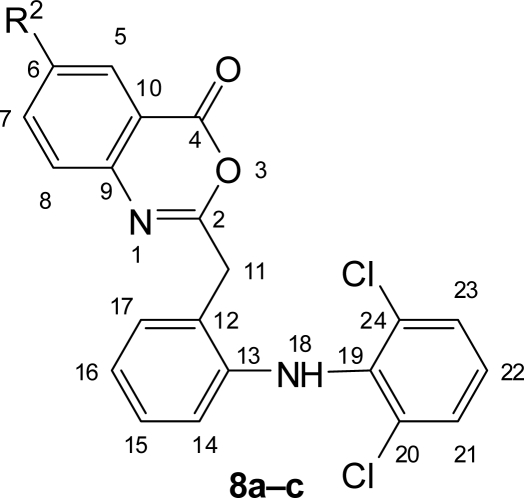
Numbering of Benzoxazinones **8a–c**

**Fig. 4.**
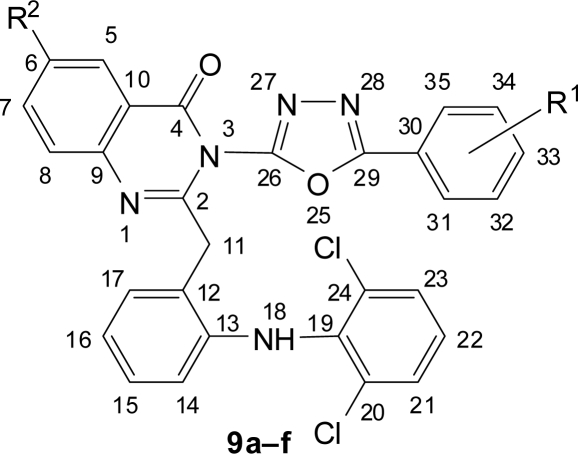
Numbering of 1,3,4-Oxadiazolyl-quinazolin-4(3*H*)-ones **9a–f**

**Fig. 5.**
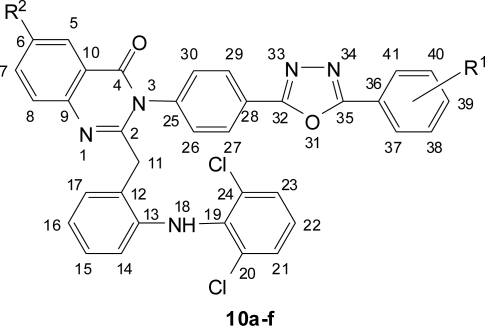
Numbering of 1,3,4-Oxadiazolyl-quinazolin-4(3*H*)-ones **10a–f**

**Fig. 6.**
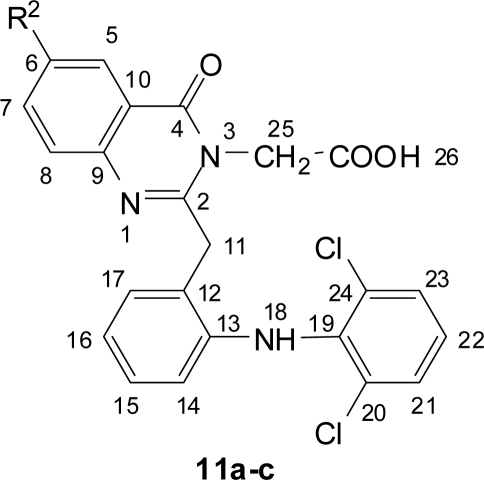
Numbering of Quinazolin-4(3*H*)-ones **11a–c**

**Fig. 7.**
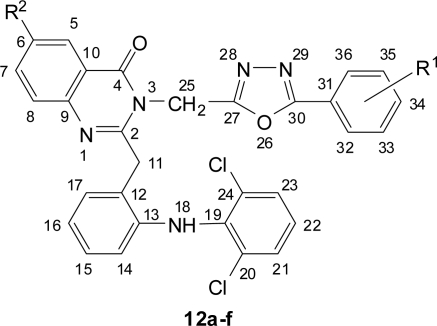
Numbering of 1,3,4-Oxadiazolyl-quinazolin-4(3*H*)-ones **12a–f**

**Sch. 1.**
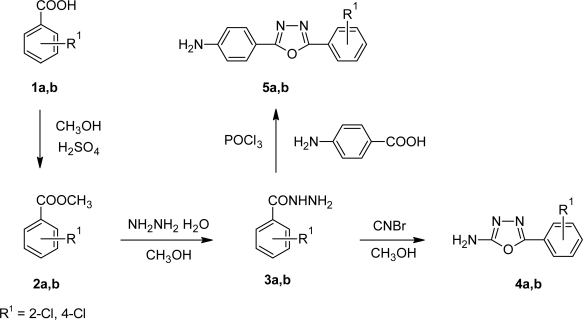


**Sch. 2.**
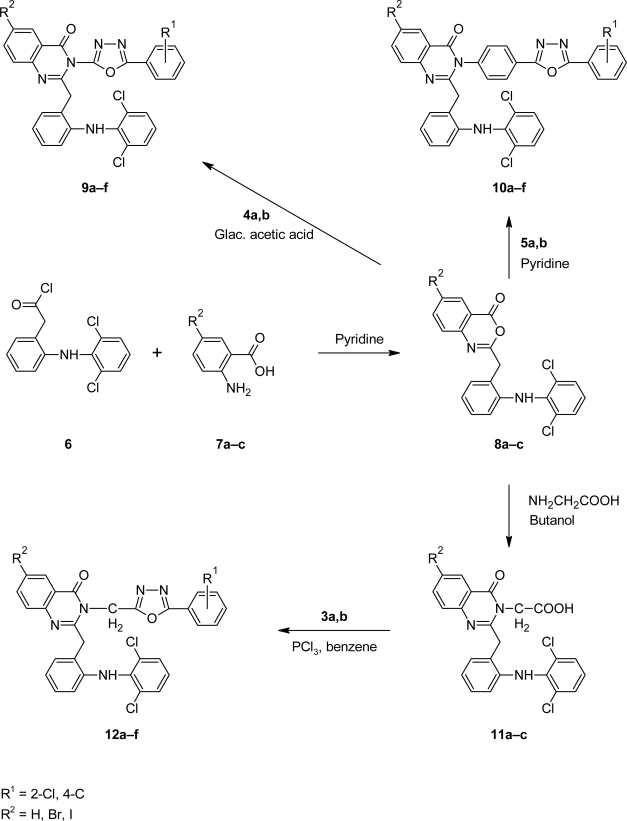


**Tab. 1.** Antibacterial activity (MBC, μg/ml) of compounds **4a,b**, **5a,b**, **9a–f**, **10a–f** and **12a–f**.

**Comp.**	**R^1^**	**R^2^**	**Gram positive bacteria**		**Gram negative bacteria**	

			***S. aureus* MTCC 96**	***S. pyogenes* MTCC 442**	***E. coli* MTCC 443**	***P. aeruginosa* MTCC 1688**
**4a**	2-Cl	–	250	250	50	250
**4b**	4-Cl	–	200	250	250	500
**5a**	2-Cl	–	100	150	250	200
**5b**	4-Cl	–	200	250	500	500
**9a**	2-Cl	H	250	200	500	500
**9b**	2-Cl	Br	100	250	200	250
**9c**	2-Cl	I	500	500	100	250
**9d**	4-Cl	H	200	250	200	250
**9e**	4-Cl	Br	250	250	250	150
**9f**	4-Cl	I	500	1000	250	200
**10a**	2-Cl	H	150	250	500	500
**10b**	2-Cl	Br	500	1000	250	500
**10c**	2-Cl	I	100	150	150	500
**10d**	4-Cl	H	500	500	200	250
**10e**	4-Cl	Br	250	250	150	250
**10f**	4-Cl	I	62.5	150	100	250
**12a**	2-Cl	H	500	500	250	250
**12b**	2-Cl	Br	500	500	100	50
**12c**	2-Cl	I	50	250	125	200
**12d**	4-Cl	H	500	500	150	200
**12e**	4-Cl	Br	500	250	100	250
**12f**	4-Cl	I	200	200	50	150
**Ampicillin**	–	–	250	100	100	100

**Tab. 2.** Antifungal activity (MFC, μg/ml) of compds. **4a,b**, **5a,b**, **9a–f**, **10a–f** and **12a–f**.

**Comp.**	**R^1^**	**R^2^**	**Fungal species**		
			***C. albicans* MTCC 227**	***A. niger* MTCC 282**	***A. clavatus* MTCC 1323**
**4a**	2-Cl	–	250	1000	1000
**4b**	4-Cl	–	>1000	>1000	>1000
**5a**	2-Cl	–	500	500	500
**5b**	4-Cl	–	500	>1000	>1000
**9a**	2-Cl	H	500	250	250
**9b**	2-Cl	Br	500	500	500
**9c**	2-Cl	I	500	500	>1000
**9d**	4-Cl	H	500	250	250
**9e**	4-Cl	Br	500	500	500
**9f**	4-Cl	I	250	200	200
**10a**	2-Cl	H	>1000	>1000	>1000
**10b**	2-Cl	Br	250	500	>1000
**10c**	2-Cl	I	>1000	>1000	>1000
**10d**	4-Cl	H	500	>1000	>1000
**10e**	4-Cl	Br	500	>1000	>1000
**10f**	4-Cl	I	250	500	500
**12a**	2-Cl	H	>1000	500	>1000
**12b**	2-Cl	Br	250	500	250
**12c**	2-Cl	I	100	200	200
**12d**	4-Cl	H	>1000	>1000	>1000
**12e**	4-Cl	Br	500	>1000	500
**12f**	4-Cl	I	200	>1000	500
**Griseofulvin**	–	–	500	100	100
